# Editorial: Community series - liver fibrosis and MAFLD: from molecular aspects to novel pharmacological strategies, volume II

**DOI:** 10.3389/fmed.2024.1371720

**Published:** 2024-02-02

**Authors:** Ana Sandoval-Rodriguez, Aldo Torre-Delgadillo, Juan Armendariz-Borunda

**Affiliations:** ^1^Institute of Molecular Biology in Medicine and Gene Therapy, Centro Universitario de Ciencias de la Salud, University of Guadalajara, Guadalajara, Jalisco, Mexico; ^2^Hepatology and Liver Transplantation Unit, Department of Gastroenterology, Instituto Nacional de Ciencias Médicas y Nutrición Salvador Zubirán (INCMNSZ), Mexico City, Mexico; ^3^Tecnologico de Monterrey, School of Medicine and Health Sciences, Guadalajara, Mexico

**Keywords:** liver fibrosis, MAFLD, MASLD, steatotic liver disease, non-alcoholic steatohepatitis (NASH)

In this consecutive second issue of Frontiers in Gastroenterology editors Armendariz-Borunda, Sandoval-Rodriguez, and Torre-Delgadillo examine new challenges in terminology, from MALFD to Steatotic Liver Disease and the new MASLD argot, retrieved from several manuscripts from 2021 to late 2023, including perspectives, systematic reviews and original research in clinical scenarios to address novel insights in the all-time important fibrosis and the new epidemic-like Metabolic Associated Steatotic Liver Disease (1).

From Thailand, Saokaew et al. present a systematic review of the effect of telemedicine in 285 patients with obesity and NAFLD in ALT, AST, TG, HDL-c levels, and body mass index outcomes. Interestingly, compared with usual care, in mostly urban populations, telemedicine significantly reduced the AST and ALT levels, indicating that some benefits can be obtained using this approach for the follow-up and treatment of patients in geographical areas where usual care is not available. This study also reminds us that even an affordable text message, telephone call, or online class can have potential benefits for the health of participants (Saokaew et al.). However, the weakness of Saokaew's review is that their metanalysis includes only three studies. In another manuscript from South Korea, the authors point out that Helicobacter pylori is a vastly prevalent bacteria that not only causes gastritis, peptic ulcers, and gastric cancer but is also linked to metabolic syndrome and cardiovascular diseases, especially arterial stiffness. Using the logistic regression model, Choi et al. conclude that Hp infection additively increases the risk of arterial stiffness in subjects with NAFLD/MAFLD, which is not surprising since there is a link between Hp infection, NAFLD, and the pathogenesis of insulin resistance and proinflammatory conditions (Choi et al.). Since the term NAFLD was coined more than four decades ago, it has changed twice, in 2020 and 2023, to highlight the importance of metabolic alterations in its pathophysiology (2, 3). In this manuscript, authors use both criteria to analyze 2,357 subjects included in an NAFLD cohort diagnosed by ultrasound and 3,195 subjects in MAFLD analysis. Regardless of the criteria, male patients had significantly more atherosclerosis risk factors predominant in NAFLD/MAFLD. In both analyses, Hp infection was independently associated with arterial stiffness as well as, a high FIB-4 index and a measurement of fibrosis (Choi et al.).

In an influential review, Falamarzi et al. summarize the role of FGF21 in liver diseases, focusing on NAFLD, AFLD, and HCC. FGF21 and its analogs have been tested to treat these pathologies due to their role in inducing beta-oxidation, improving insulin sensitivity, and decreasing VLDL. FGF21 has also been proposed as a biomarker for NAFLD since its increase correlates with the amount of hepatic fatty acids. Therefore, its use as a delay agent not only for NAFLD but also for AFLD and HCC seems opposite; however, its protective role has been validated in knockout FGF21 mice that showed enhanced inflammation and steatohepatitis. FGF21 administration in experimental alcoholic liver disease demonstrated positive effects in preventing fatty liver progression serum lipid profiles and reduction in oxidative stress. FGF21 analog administration in mice models and human metabolic diseases showed anti-inflammatory, anti-diabetic, and hypolipidemic effects, reversing hepatic steatosis. Especially in HCC, overexpression of FGF21 delays adenoma development, however, it accelerates the progression of tumors to HCC due to FGFR1 interaction. FGF21 decrement has a role in progression to HCC, inducing a microenvironment wherein inflammation, mitotic regenerating factors for hepatocytes, and fibrosis predominate. Furthermore, some genetic variants for FGF21 have been linked to MAFLD, risk behaviors (alcohol, candy, and cigar consumption), and eating habits connecting these variations to obesity and alcohol dependence, diseases in which steatosis pathophysiology is important. In conclusion, FGF21 is a molecule that should be in our minds when thinking about metabolic diseases concerning the liver (Falamarzi et al.).

Griffett and Burris elegantly review how LXR inverse agonist molecules enabled the recruitment of corepressors that silence *de novo* lipogenesis (DNL)-related genes act in dyslipidemia and MAFLD. As nuclear receptors, LXRs are master regulators of lipid and cholesterol metabolism, intricately involved in the regulation of DNL in the liver, meaning they are very interesting potential drug targets. The use of LXR agonists displays anti-atherogenic properties by increasing cholesterol efflux from peripheral tissues; however, this can also lead to outcomes in hepatic steatosis. Since LXRα and LXRβ can recruit either coactivators or corepressors, this research group has developed LXR ligands (SR9238 and SR9243) that selectively enhanced the ability of LXRs to recruit corepressors like NCoR ID1 and NCoR ID2 peptides and in consequence decreases the expression of *Fasn, Srebf1c*, and *Scd1*, specifically in the liver not affecting reverse cholesterol transport (RCT) in peripheral tissues. SR9238 administration decreased the expression of genes encoding DNL enzymes and in consequence diminished hepatic steatosis in DIO mice, ob^−^/ob^−^ mice fed with a diet high in cholesterol, fructose, and trans-fat, mice under Lieber-DiCarli diet, ASH rodent model using chronic ethanol consumption plus “binge” ethanol doses, and even in a dual model of mice fed with high cholesterol/trans-fat/fructose and ethanol. Interestingly, these compounds decrease plasma LDL-C, with potential efficacy for hypercholesterolemia. Due to their beneficial effects, some other LXR inverse agonists with similar pharmacological profiles have been developed by other researchers and pharma companies, reaching phase I clinical trials for the treatment of severe dyslipidemia—TLC-2716- (Griffett and Burris).

In another contribution to this issue, researchers from Singapore use data from the National Health and Nutrition Examination Survey (1999–2018) of the general non-institutionalized population. Their results show that 30% of NAFLD patients had concomitant depression. Using AASLD-NAFLD criteria and defining depression as the use of antidepressants or ≥10 scores on the Patient Health Questionnaire-9 (PHQ-9); in multivariate analysis older age, female gender, diabetes, higher BMI, hypertension, being Hispanic or Caucasian; resulted in an increased risk of depression amongst individuals with NAFLD. The impact of suffering depression in NAFLD patients—adjusting for age, gender, race, BMI, and diabetes- is linked to complications like CVD and stroke, and a 50% increased risk of mortality (no significance for CVD mortality but weighted for cancer-related mortality). These complications are reduced in depression-treated NAFLD patients, compared to untreated depression. The link between depression and NAFLD comes from insulin resistance onset, which can alter insulin signaling in the brain, overexpression of TNFa, IL-6, and monoamine oxidase-A that may also potentiate mood disorders and the presence of concomitant diabetes and obesity, resulting in oxidative stress and inflammation (Ng et al.). Other studies have also found an association between depression and NAFLD (4) reminding us of the importance of including periodic screening for depression in clinical practice guidelines and holistic care for patients with NAFLD. An innovative study by Meyer et al. approaches an ancient issue in NAFLD dynamics that is involved with fibrosis development. Using data from seven published clinical studies in which patients had biopsy-proven NAFLD or NASH, they developed a computational continuous-time Markov chain model that claims to capture the well-known clinical heterogeneity of fibrosis progression to provide alertness for clinical trial design. As a proof of principle, they applied the model to quantify pioglitazone effects on fibrosis progression. The advantages of continuous-time Markov chain models are the fact that they are probabilistic and time-independent. In this particular model, five potential states represent each stage of fibrosis (according to Kleiner or Brunt scoring), and progressors move through the stages of fibrosis with a probability of progression or regression that is independent of how long the subject was in that fibrosis score. After data fitting and sensitivity analysis, the authors assessed how an intervention, such as pioglitazone, impacts the forward and reverse model parameters. Using the model, the results indicate that pioglitazone slows disease progression and reverses fibrosis, based on the faster transition to lower fibrosis scores, showing good fitting of observed and predicted data. Since fibrosis progression in NAFLD and NASH is not well characterized, this model could predict, given an initial distribution, a percentage of progressors from non-progressors, also this model estimates that intervention at early stages of fibrosis has more possibilities to improve fibrosis score. Regarding the aim of impact clinical trial design, employing this model suggests a sample of 65 patients to detect a change of 0.5 in fibrosis score, supposing drug effects similar to pioglitazone, using 80% power. Taking into account variability in sample size and location, and pathologist observation. This model promises to become more powerful including more data from clinical studies or combining with bridging a quantitative systems pharmacology (QSP) model that connects mechanistic drug effects to clinical outcomes (Meyer et al.).

Two reviews in this Frontiers in Medicine issue, elegantly cover fibrosis from the exploration of multi-drug treatment to animal models. Dong et al. discuss the therapeutic strategy of multi-drug combination for MAFLD-related liver fibrosis (Dong et al.). It is now accepted that liver fibrosis can be reversed by eliminating the etiological agent, but not all causes of chronic liver damage can be successfully removed, especially for MAFLD-related liver fibrosis. Approaches adopting single lifestyle interventions have not controlled the prevalence of MAFLD and in consequence, other therapeutic strategies need to be added. Approximately 40% of MAFLD patients are non-obese. For this cohort, the pharmacological treatment for MAFLD and its related liver fibrosis might be beneficial, including drugs that act on lipogenesis and fat accumulation, antioxidants, and agents that act in inflammation and extracellular matrix accumulation. However, few treatments to date have reached acceptable outcomes when assessed by liver biopsy. The exploration of multi-drug combination therapies is therefore appropriate since various mechanisms of MAFLD and fibrosis progression can be targeted. The authors analyze the possibility of combining drugs including GLP-1 receptor agonists, acetyl-CoA carboxylase inhibitors, FXR agonists, PPARs modulators, endothelial cell modulators, and natural compounds like sesquiterpene ketone, hydroxytyrosol, and vitamin E. In conclusion, since MAFLD has become the primary cause of liver fibrosis and one of the most common indications for liver transplant, it has become essential to explore multi-drug combinations targeting diverse fibrosis regression mechanisms and MAFLD pathophysiology.

Concerning fibrosis animal models, Wu et al. review the applications, advantages, and disadvantages, to select the appropriate model according to the research purpose. Independently of the five forms - chemical, dietary, surgical, transgenic, and immune – that are used to induce liver fibrosis, the molecular mechanisms are involved in largely similar pathways, including oxidative stress, inflammation, alteration in hepatic lipid metabolism, hepatocyte injury, HSC activation, and increased production of ECM (Wu et al.). One of the major decisions involved with this is the selection of species as the basis of space availability and modeling time. For example, alcohol is easily accessible, but *ad libitum* consumption time goes up to 70 weeks to produce insufficient liver injury to cause fibrotic or cirrhotic lesions. While CCl4 i.p. intoxication is a simple method of short-duration induction and causes significant hepatic pathological changes. Here, the authors also recapitulate models that include body-composition modifications where animals become obese or develop insulin resistance, reflecting the pathophysiology of MASLD and related metabolic alterations. An exception to this is the choline-deficient L-amino-defined diet-induced liver fibrosis model and the methionine-choline deficient-induced liver fibrosis model, where animals do not gain weight and lack characteristics of metabolic syndrome; however, steatohepatitis and fibrosis can be induced within shorter time. Transgenic and immune induction methods require specialized facilities and personnel, meaning these models are expensive and less used. Lately, a combination of methods is a common and effective way to model actual situations such as the high prevalence of MASLD and chronic or binge ethanol consumption. Each model has its drawbacks and advantages, and the development of such a variety of models through the years has led to a current set of experimental models that fit almost any purpose and requirement.

This Research Topic also includes a final contribution by Sergi, examining the relationship between MASLD and aspartame. Aspartame—methyl L-α-aspartyl-L-phenylalaninate- is a sweetener labeled in 2023 as a possible carcinogenic compound that can be offered as an anti-steatogenic food component. Sergi reminds us that prolonged aspartame administration leads to hepatic fibrosis, upregulating Tgfb1, Col1A1, and aSma; reducing the activation of Nrf2 and increased lipid peroxidation, which triggers NLRP3 (NOD-like receptor containing protein 3) inflammasome activation and p53 induction. Aspartame reduces PGC1α, a transcriptional coactivator, that upregulates mitochondrial biogenesis, oxidative phosphorylation, respiratory capacity, and fatty acid β-oxidation. Even though these data were obtained in experimental animals, Sergi argues that the results may be relevant in humans and linked to liver cancer through NLRP3 inflammasome activation (Sergi). Artificially sweetened soft drinks have been modeled as a healthier alternative to other drinks, however, the use of aspartame, saccharine, acesulfame-K, sucralose, and neotame is controversial, since some studies found them to be linked to some neurological effects. These perspectives motivate the realization of clinical trials to confront this theoretical conjecture, a good reminder of the importance of basic research and its translation to clinical scenarios.

This Research Topic includes contributions from experts in this area, including original research and review articles that address MAFLD from basic and clinical perspectives and update important aspects of this research. More than ever, this research is vital for treating actual and silent diseases.

## Author contributions

AS-R: Conceptualization, Writing—original draft, Writing—review & editing. AT-D: Writing—original draft, Writing—review & editing. JA-B: Writing—original draft, Writing—review & editing, Conceptualization.
